# Identification of a mycobacterial hydrazidase, an isoniazid-hydrolyzing enzyme

**DOI:** 10.1038/s41598-023-35213-5

**Published:** 2023-05-20

**Authors:** Arata Sakiyama, Chaogetu Saren, Yukihiro Kaneko, Ken-Ichi Oinuma

**Affiliations:** 1 Department of Bacteriology, Osaka City University Graduate School of Medicine, Abeno-ku, Osaka, Japan; 2 Department of Bacteriology, Osaka Metropolitan University Graduate School of Medicine, Abeno-ku, Osaka, Japan; 3 Research Center for Infectious Disease Sciences, Osaka Metropolitan University Graduate School of Medicine, Abeno-ku, Osaka, Japan

**Keywords:** Hydrolases, Bacterial infection, Antimicrobial resistance

## Abstract

There exists decades-old evidence that some mycobacteria, including *Mycobacterium avium* and *Mycobacterium smegmatis*, produce hydrazidase, an enzyme that can hydrolyze the first-line antitubercular agent isoniazid. Despite its importance as a potential resistance factor, no studies have attempted to reveal its identity. In this study, we aimed to isolate and identify *M. smegmatis* hydrazidase, characterize it, and evaluate its impact on isoniazid resistance. We determined the optimal condition under which *M. smegmatis* produced the highest amount of hydrazidase, purified the enzyme by column chromatography, and identified it by peptide mass fingerprinting. It was revealed to be PzaA, an enzyme known as pyrazinamidase/nicotinamidase whose physiological role remains unknown. The kinetic constants suggested that this amidase with broad substrate specificity prefers amides to hydrazides as a substrate. Notably, of the five tested compounds, including amides, only isoniazid served as an efficient inducer of *pzaA* transcription, as revealed by quantitative reverse transcription PCR. Moreover, high expression of PzaA was confirmed to be beneficial for the survival and growth of *M. smegmatis* in the presence of isoniazid. Thus, our findings suggest a possible role for PzaA, and other hydrazidases yet to be identified, as an intrinsic isoniazid resistance factor of mycobacteria.

## Introduction

Infectious diseases caused by mycobacteria (members of the genus *Mycobacterium*) pose a global threat to human health. A particularly serious concern is the tuberculosis (TB) epidemic caused by *M. tuberculosis* (Mtb), which kills more than 1 million people annually^1^. Moreover, the incidence of infection by non-tuberculous mycobacteria (NTM), including the *M. avium* complex, mainly composed of *M. avium* and *M. intracellulare*, is increasing globally. NTM exist in various environments, including the living environments of humans, and can cause chronic pulmonary infections not only in immunosuppressed individuals but also in otherwise healthy individuals^2,3^. Treatment of mycobacterial infections is challenging and generally requires the long-term use of multiple antimicrobials. TB is typically treated with the first-line drugs isoniazid (INH), rifampicin, ethambutol, and pyrazinamide for 2 months and with INH and rifampicin for an additional 4 months^4^. The treatment of TB caused by multidrug-resistant Mtb, which is known to show resistance to both INH and RFP, poses a greater challenge^4^ than drug-sensitive TB, as is true for infections caused by naturally INH-resistant NTM^2^.

INH is a prodrug that functions after its activation by the mycobacterial catalase-peroxidase KatG. KatG-activated INH undergoes a coupling reaction with NADH, and the resulting INH-NAD adduct inhibits an enoyl reductase named InhA, which is involved in the biosynthesis of mycolic acids, a major component of the mycobacterial cell wall. INH mediates its effect by disturbing the mycobacterial cell wall and killing Mtb^5,6^*.* In the past several decades, the mechanism underlying INH resistance in Mtb has been the subject of intensive research. In approximately 80% of cases, INH resistance in Mtb occurs owing to mutations in either *katG* or *inhA*. Mutations in *acpM* and *kasA*, which are related to mycolic acid biosynthesis, have also been identified as alternative causes of INH resistance in Mtb.

**Figure 1:**
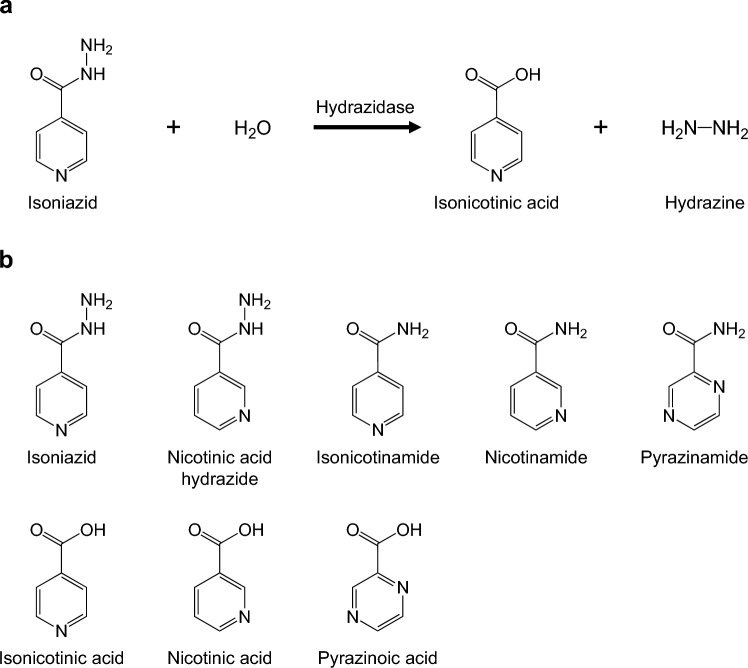
Reaction scheme of isoniazid hydrolysis by hydrazidase (**a**) and structures of isoniazid and its analogs used in this study (**b**).

Although INH is considerably potent against Mtb and some NTM, including *M. kansasii*, it is substantially less effective against other mycobacteria, including *M. avium* complex, even though most, if not all, of these mycobacteria have similar mycolic acid synthetic mechanisms. In contrast to the INH resistance mechanisms in Mtb, which have been intensively explored, limited attention has been paid to the mechanisms underlying the intrinsic INH resistance in NTM, leaving many possible hypotheses unexplored. One example of such hypotheses is the one involving INH-degrading enzymes that might confer resistance on NTM by detoxifying INH. According to studies conducted by Toida in the 1950s–1960s^7–9^, some strains of *M. avium* produce an INH-hydrolyzing enzyme, named hydrazidase, in response to exposure to INH and hydrolyze this agent into isonicotinic acid and hydrazine (Fig. 1a), which are compounds that are less toxic to this bacterium than INH. Notably, an INH-resistant strain, prepared by repeatedly subculturing a susceptible strain in media supplemented with INH, showed 27-fold higher INH-degrading activity than the original strain^7^. These results suggest that hydrazidase might play a role in INH resistance in *M. avium*, although no concrete evidence to support this theory is available. Additionally, another group reported a similar INH-degrading activity in *M. smegmatis*^10^. The authors first obtained INH-resistant strains of *M. smegmatis* by serial passage in media with increasing INH concentration and then compared their characteristics with those of the original susceptible strain. Both INH-resistant and -susceptible strains were found to be able to degrade INH, with similar INH degradation rates. Moreover, the resistant strain grew in the presence of INH without destroying significant amounts of it. Thus, the authors concluded that resistance does not depend on INH destruction. However, it must be noted that this study mainly focused on the causes of the acquired resistance of *M. smegmatis*, and whether or not this enzyme is contributing to the relatively high basal INH resistance of *M. smegmatis* was not addressed.

To the best of our knowledge, whether or not these INH-hydrolyzing enzymes contribute to the intrinsic INH resistance of NTM is still an open question. No progress has been reported regarding these enzymes in the last several decades, and their identity remains unclear. In this study, we aimed to isolate and identify a hydrazidase from *M. smegmatis*. We also investigated the conditions under which the identified enzyme is expressed in *M. smegmatis* cells and determined its enzymatic properties. Additionally, we explored the possibility that hydrazidases are involved in the inherent INH resistance of mycobacteria, which yielded new data supporting this hypothesis.

## Results

### Confirmation of INH-hydrolyzing activity in *M. smegmatis* and optimization of growth conditions for maximum enzyme production

**Figure 2:**
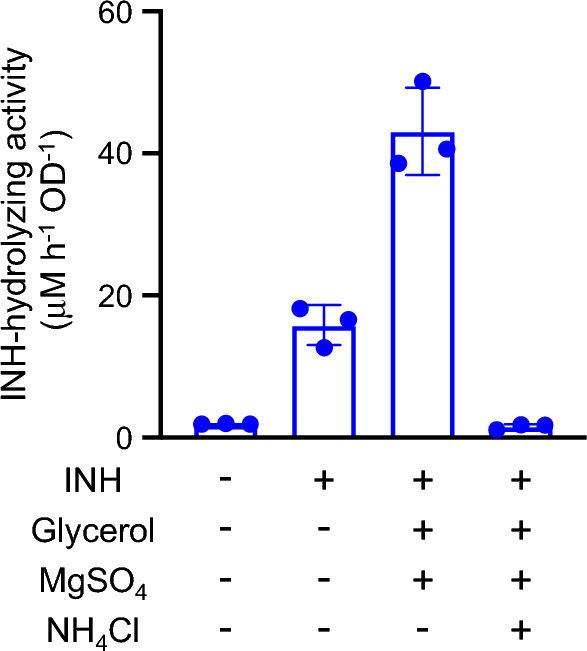
Effect of synthetic media additives on the isoniazid (INH)-hydrolyzing activity of *Mycobacterium smegmatis*. *M. smegmatis* MC^2^155 cells grown in 7H9 medium for 40 h were transferred to synthetic media containing the indicated components and incubated for 8 h. The activities of resting cells were normalized by the optical density (OD) of the cell suspension measured at 600 nm. Dots and bars indicate individual values and the mean of triplicate cultures. Error bars indicate standard deviations.

We first tested whether *M. smegmatis* MC^2^155 cells show INH-hydrolyzing activity like *M. smegmatis* NRC 43006, in which such activity was previously observed^10^. MC^2^155 cells cultured in 7H9 for 48 h indeed hydrolyzed INH as confirmed by the formation of isonicotinic acid and hydrazine, and the rate measured by monitoring the formation of isonicotinic acid (normalized by optical density [OD] of the cell suspension measured at 600 nm) was 0.711 (standard deviation [SD] 0.226) μM h^−1^ OD^-1^. When we added 1 mM INH at 40 h of growth, the activity increased by 1.8-fold (1.25 [SD 0.217] μM h^−1^ OD^−1^) compared to that in the culture without the addition of INH, suggesting that the production of the responsible enzyme is inducible by INH. We then attempted to optimize the induction and considered the dependence of the activity of this enzyme on the growth media composition. In this experiment, we used a two-step cultivation method, in which MC^2^155 cells were first cultivated in 7H9 for 40 h and then transferred to M9-based synthetic media with different components and cultivated for another 8 h to induce enzyme production. These synthetic media were a phosphate buffer composed of 42 mM Na_2_HPO_4_, 22 mM KH_2_PO_4_, and 9 mM NaCl, and contained none, one, or more of the following chemicals: INH (1 mM), glycerol (22 mM [0.2%]), MgSO_4_·7H_2_O (2 mM), and NH_4_Cl (19 mM [0.1%]). Although the activity was considerably low when the medium with no supplement was used (1.95 [SD 0.0404] μM h^−1^ OD^−1^), it increased 8.1-fold (15.8 [SD 2.83] μM h^−1^ OD^−1^) when the medium was supplemented with INH (Fig. 2). Supplementation with glycerol and MgSO_4_ further enhanced the activity (43.1 [SD 6.16] μM h^−1^ OD^−1^). Conversely, the addition of NH_4_Cl to the synthetic medium containing INH, glycerol, and MgSO_4_ suppressed the activity (1.57 [SD 0.365] µM h^−1^ OD^−1^) to the level of uninduced cells. Hereafter we refer to this synthetic medium containing glycerol and MgSO_4_ but not a nitrogen source as “nitrogen-depleted M9”.

Next, to optimize the culture conditions, the influence of induction duration and INH concentrations was investigated. When the induction duration was compared among five time points from 1 to 24 h (1, 3, 6, 12 and 24 h), the activity peaked at 6 h (58.9 µM h^−1^ OD^−1^) and then stabilized. INH concentrations were tested at seven values (0.0625, 0.125, 0.25, 0.5, 1, 2, and 4 mM), and 0.5 mM was found to be the optimal concentration (64.2 [SD 11.9] μM h^−1^ OD^−1^).

Additionally, we investigated whether the addition of nitrogen sources other than NH_4_Cl suppresses the INH-hydrolyzing activity. In this experiment, we cultivated MC^2^155 cells in the presence of one of the following compounds (0.1% each) under otherwise optimal conditions: ammonium sulfate, ammonium acetate, sodium nitrate, urea, alanine, asparagine, aspartic acid, glutamine, glutamic acid, arginine, and bovine serum albumin (BSA). All the compounds, except for sodium nitrate, arginine, and BSA, showed a strong suppressive effect on the INH-hydrolyzing activity. The effects of ammonium sulfate and ammonium acetate were particularly strong, decreasing the activity to 1.28 (SD 0.292) μM h^−1^ OD^−1^ and 1.96 (SD 0.713) μM h^−1^ OD^−1^, respectively. The activities of cells after incubation with urea, alanine, asparagine, aspartic acid, glutamine, or glutamic acid fell within the range of 3.17–11.7 µM h^−1^ OD^−1^. Overall, these observations demonstrate that nitrogen depletion is a key factor determining the INH-hydrolyzing activity of *M. smegmatis*.

### Purification and identification of the INH-degrading enzyme from* M. smegmatis* MC^2^155

Cell extracts of strain MC^2^155, prepared from 2 L of the optimized medium to use as a purification starter, showed the isonicotinic acid-forming activity at 0.0435 (SD 0.000833) μmol min^−1^ mg^−1^. Moreover, by using ammonium sulfate precipitation and four types of column chromatography, the enzyme was successfully purified 48.5-fold with a recovery rate of 1.6% (Table 1). As expected, the purified preparation showed not only the isonicotinic acid-forming activity at 2.11 (SD 0.0140) μmol min^−1^ mg^−1^ but also a hydrazine-forming activity, although the latter activity was calculated to be 1.26 (SD 0.502) μmol min^−1^ mg^−1^, a value slightly lower than the former. In theory, the INH-hydrolyzing reaction yields the same amount of hydrazine and isonicotinic acid, and the formation rates of these products should be the same. The observed difference between the formation rates may reflect the difference in the accuracy between the two detection methods or the fact that hydrazine is more unstable than isonicotinic acid.

**Table 1:** Summary of hydrazidase purification from *Mycobacterium smegmatis* MC^2^155.

Purification steps	Protein	Specific activity	Total activity	Yield	Purification fold
	(mg)	(μmol min^−1^ mg^−1^)	(μmol min^−1^)	(%)	
Cell-free extracts	83.7	0.0435 (0.000833)	3.64	100	1
40–60% AS precipitate	24.2	0.0775 (0.00753)	1.88	52	1.78
HiTrap Q fraction	3.20	0.400 (0.0494)	1.28	35	9.20
HiTrap Butyl fraction	0.280	1.77 (0.0248)	0.496	14	40.7
Superdex 200 fraction	0.124	2.06 (0.0396)	0.255	7.0	47.4
Resource Q fraction	0.0270	2.11 (0.0140)	0.0570	1.6	48.5

Hydrazidase activity was determined using the UPLC-based method that detected isonicotinic acid. The reaction time was 3 h for all samples. The values shown as specific activity are means of triplicate assays. Standard deviations are shown in parentheses. AS, ammonium sulfate.

**Figure 3:**
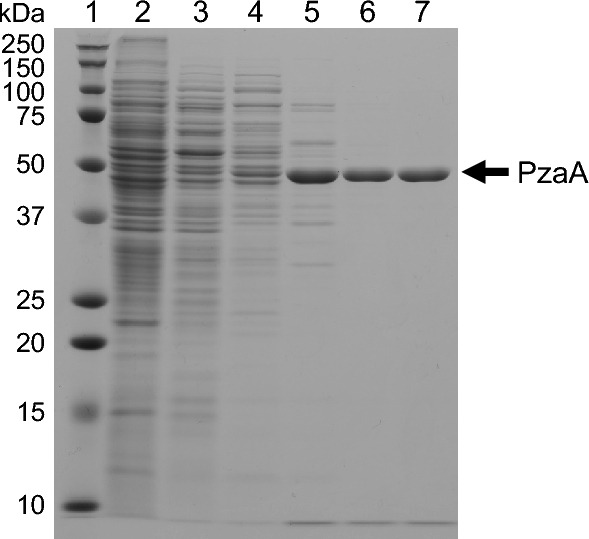
Purification of the isoniazid (INH)-hydrolyzing enzyme from *Mycobacterium smegmatis* MC^2^155. A cropped image of a Coomassie brilliant blue-stained sodium dodecyl sulfate–polyacrylamide gel electrophoresis gel is shown. The original uncropped image is presented in Supplementary Fig. S1. The arrow indicates the purified enzyme. Lane 1, molecular weight markers (the molecular mass of each marker in kilodaltons is indicated to the left of the gel); lane 2, cleared cell extract; lane 3, 40–60% ammonium sulfate precipitate; lane 4, pooled PzaA-containing fractions from HiTrap Q HP column chromatography; lane 5, pooled PzaA-containing fractions from HiTrap Butyl HP column chromatography; lane 6, pooled PzaA-containing fractions from Superdex 200 column chromatography; lane 7, pooled PzaA-containing fractions from Resource Q column chromatography.

In addition, using sodium dodecyl sulfate–polyacrylamide gel electrophoresis (SDS-PAGE), the purity and the molecular mass of the enzyme were found to be 99% or higher and approximately 49 kDa (Fig. 3 and Supplementary Fig. S1). We then performed matrix-assisted laser desorption/ionization-time of flight-mass spectrometry to identify the enzyme using peptide mass fingerprinting. The MASCOT search identified an amidase named PzaA (UniProtKB accession number: I7FF89) as the most probable candidate. The recovery rate was 64% (Supplementary Data S1). This enzyme is known to hydrolyze pyrazinamide and nicotinamide, but its physiological role remains unknown. To confirm that PzaA is the enzyme responsible for the observed INH-hydrolyzing activity, we constructed Δ*pzaA*, a *pzaA* deletion mutant of *M. smegmatis* MC^2^155. As expected, Δ*pzaA* did not exhibit INH-hydrolyzing activity even under the optimized conditions. To further confirm the functionality of *pzaA*, we complemented Δ*pzaA* with a PzaA expression plasmid pMV261-*pzaA*. Cell-free extracts of the complemented strain (Δ*pzaA* pMV261-*pzaA*), grown in a 7H9 broth for 40 h, hydrolyzed INH at 0.0275 (SD 0.00467) μmol min^−1^ mg^−1^ (calculated from the isonicotinic acid formation rate), whereas extracts from a control strain containing an empty vector (Δ*pzaA* pMV261) did not show any detectable activity. These results demonstrate, for the first time, that PzaA is involved in the degradation of INH by *M. smegmatis*.

### PncA is incapable of hydrolyzing isoniazid

In addition to PzaA, *M. smegmatis* has another nicotinamidase/pyrazinamidase named PncA (UniProtKB accession number: I7FN79). While PzaA belongs to the amidase signature family, PncA is classified in the isochorismatase family. These two enzymes are structurally and phylogenetically distinct, as is evident from their difference in the overall amino acid sequence length (468 amino acids for PzaA versus 183 amino acids for PncA). Nevertheless, these enzymes likely have similar substrate specificity because they both hydrolyzed pyrazinamide and nicotinamide at comparable rates when expressed in *M. bovis* BCG^11^. Therefore, we speculated that PncA might also be capable of hydrolyzing INH. To test this hypothesis, we prepared two Δ*pzaA* strains containing an acetamide-inducible PncA expression plasmid (pMyC-*pncA*) or an empty vector (pMyC) and used them to evaluate the INH-hydrolyzing activity of PncA. Cell-free extracts of the PncA-expressing strain, grown in 7H9 and induced with acetamide, hydrolyzed nicotinamide and pyrazinamide at 0.0790 (SD 0.00459) μmol min^−1^ mg^−1^ and 0.0507 (SD 0.000867) μmol min^−1^ mg^−1^, respectively. These rates are 51 to 79-fold higher than those of the empty vector control strain (0.001 [SD 0.0008] μmol min^−1^ mg^−1^; the rates were the same for both substrates). These observations demonstrate the successful expression of PncA in the Δ*pzaA* pMyC-*pncA* cells. However, neither of these strains showed any detectable activity against INH, indicating that PncA is incapable of hydrolyzing INH.

### Enzymatic characteristics of PzaA

We performed steady-state kinetics experiments, including an assessment of substrate specificity, using purified PzaA. The *V*_max_ and* K*_m_ values of the enzyme for INH, determined using the colorimetric method monitoring formation of hydrazine, were 1.51 (SD 0.140) μmol min^−1^ mg^−1^ and 1.20 (SD 0.271) mM, respectively. PzaA degraded nicotinic acid hydrazide, isonicotinamide, nicotinamide, and pyrazinamide (Fig. 1b) more effectively than it degraded INH, showing higher *V*_max_ (6.48–37.2 μmol min^−1^ mg^−1^) and/or lower *K*_m_ (0.0887–0.145 mM) values (Table 2). These findings indicate that PzaA has broad substrate specificity and is more specific for amides than for hydrazides.

**Table 2:** Kinetic constants of PzaA.

Substrate	*V*_max_	*K*_m_	*V*_max_/*K*_m_
	(μmol min^−1^ mg^−1^)	(mM)	(μmol min^−1^ mg^-1^ mM^−1^)
Isoniazid	1.51 (0.140)	1.20 (0.271)	1.26
Nicotinic acid hydrazide	23.7 (0.788)	1.26 (0.0475)	18.8
Isonicotinamide	6.48 (0.102)	0.145 (0.0203)	44.7
Nicotinamide	37.2 (0.287)	0.0887 (0.0113)	419
Pyrazinamide	11.7 (0.508)	0.143 (0.00780)	81.8

Enzyme assays with hydrazides and amides as substrates were performed using colorimetric methods that monitored the formation of hydrazine and ammonia, respectively. Lineweaver–Burk plots were used to calculate the *V*_max_ and *K*_m_ values. Data represent means of triplicate values obtained from three separate data sets. Standard deviations are shown in parentheses.

### Ability of various INH analogs to induce PzaA production

As our findings suggested that PzaA has broad signal specificity, we then determined whether INH analogs can induce the expression of PzaA similar to INH. We first tested seven INH analogs (nicotinic acid hydrazide, isonicotinamide, nicotinamide, pyrazinamide, isonicotinic acid, nicotinic acid, and pyrazinoic acid) (Fig. 1b) as potential inducers. After cells were incubated in nitrogen-depleted M9 containing one of these compounds (0.5 mM) for 30 min or 6 h, the whole-cell assay using INH as the substrate was performed to measure the isonicotinic acid-forming activity of the cells, which served as an indicator of PzaA expression level. None of these compounds induced the expression of PzaA effectively; the calculated isonicotinic acid-forming activities after incubation for 30 min and 6 h were within the ranges of 6.79–19.8 µM h^−1^ OD^−1^ and 2.49–7.49 µM h^−1^ OD^−1^, respectively, which were considerably lower than the values obtained using INH as an inducer (27.1 [SD 6.59] μM h^−1^ OD^−1^ and 59.9 [SD 7.59] μM h^−1^ OD^−1^ after 30 min and 6 h of induction, respectively) and comparable or only up to 2.2-fold higher than the values obtained without an inducer (8.86 [SD 2.34] μM h^−1^ OD^−1^ and 3.59 [SD 1.33] μM h^−1^ OD^−1^ after 30 min and 6 h of induction, respectively).

**Figure 4:**
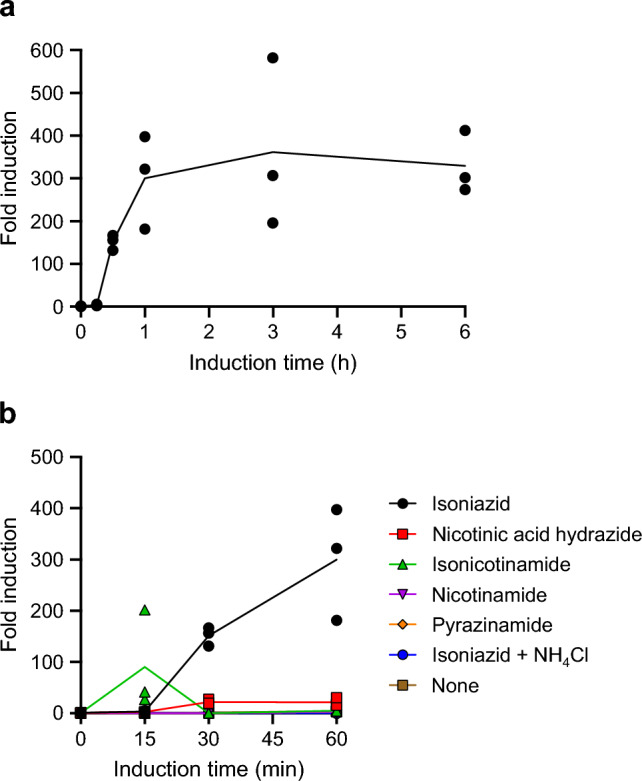
Regulation of *pzaA* transcription in *Mycobacterium smegmatis* MC^2^155 monitored using quantitative reverse transcription PCR. (**a**) Time series of *pzaA* mRNA levels in cells cultured with isoniazid (0.5 mM). (**b**) Time series of *pzaA* mRNA levels in cells cultured with various isoniazid analogs or isoniazid (0.5 mM each) with or without NH_4_Cl (0.1%). Data are expressed as fold-change of mRNA levels relative to 0-h samples. Dots and lines indicate individual values and the mean of triplicate cultures.

Next, to determine whether the induction of PzaA production was caused by the upregulation of *pzaA* transcription and to compare the abilities of INH and some of the INH analogs (nicotinic acid hydrazide, isonicotinamide, nicotinamide, and pyrazinamide) to cause such transcriptional upregulation, we observed the time course of the *pzaA* mRNA levels during the induction process with each compound. When the bacteria were cultured in a medium supplemented with INH, the mRNA level increased 300-fold in 1 h and remained stable until 6 h (Fig. 4a). This result was somewhat surprising because the fold-change of the *pzaA* mRNA levels was substantially greater than what we expected from the fold-change of INH-hydrolyzing activity between INH-induced and -uninduced cells observed in earlier experiments. We then tested the ability of nicotinic acid hydrazide, isonicotinamide, nicotinamide, and pyrazinamide to induce *pzaA* transcription. In this experiment, we monitored the *pzaA* mRNA levels only for 1 h because a longer induction with these compounds did not have any positive effects on the INH-hydrolyzing activity of cells, and all these tested compounds, except nicotinic acid hydrazide, disappear from the media within 1 h presumably by enzymatic degradation. In contrast to INH, these compounds did not stimulate *pzaA* transcription as effectively as INH, although a 90-fold induction at 15 min and a 22-fold induction at 30–60 min were observed with isonicotinamide and nicotinic acid hydrazide, respectively (Fig. 4b). To confirm that nicotinic acid hydrazide does not exert a strong effect at a later time, we determined the *pzaA* mRNA level after 6 h of induction with this compound. The mRNA level after 6 h of induction was even lower than that at the beginning of the induction (0 h). Additionally, we found that the addition of 0.1% NH_4_Cl completely inhibited the *pzaA* transcriptional induction (Fig. 4b), which explains the observation that the addition of NH_4_Cl abolished the stimulation of INH-hydrolyzing enzyme production by INH (Fig. 2). These results clearly reveal the presence of a transcriptional regulation system for *pzaA* that can be upregulated and downregulated by INH and NH_4_Cl, respectively. However, whether isonicotinamide, nicotinamide, and pyrazinamide are capable of stimulating this system remains unclear because our experiments could not distinguish between the possibilities that these amides were incapable of stimulating *pzaA* transcription and that they were capable of doing so, but transcriptional induction was rapidly abolished due to ammonia released from these amides through enzymatic hydrolysis by PzaA or other amidases. The latter possibility is especially likely in the case of isonicotinamide, considering that it transiently showed a stimulating effect at 15 min of induction. In addition, we found that isonicotinamide and nicotinamide were rapidly consumed by MC^2^155 cells in the synthetic media and they were undetectable after 30 min of cultivation. The rapid disappearance from the induction media may also contribute to the failure of these amides to induce (or keep inducing) *pzaA* transcription.

### Expression of PzaA at high levels is beneficial to the survival and growth of *M. smegmatis* in the presence of INH

**Figure 5:**
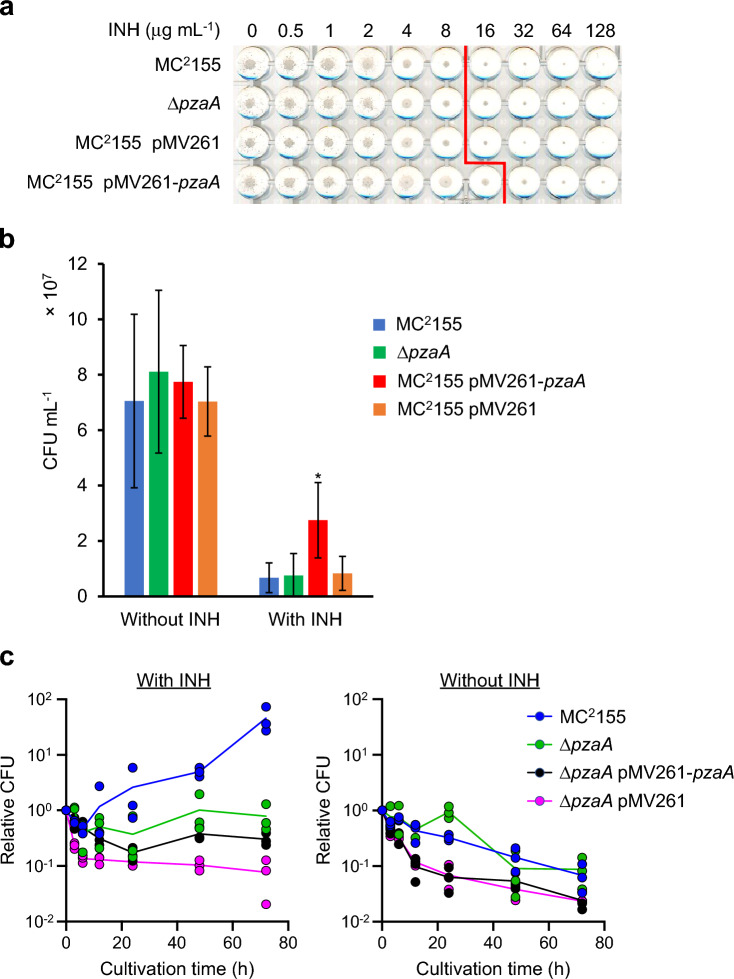
Effects of PzaA production on survival and growth of *Mycobacterium smegmatis* MC^2^155 in the presence of isoniazid (INH). MC^2^155 and its derivatives (a *pzaA* deletion mutant [Δ*pzaA*], strains with the PzaA expression plasmid [MC^2^155 pMV261-*pzaA* and Δ*pzaA* pMV261-*pzaA*], and empty vector control strains [MC^2^155 pMV261 and Δ*pzaA* pMV261]) were subjected to susceptibility tests by the broth microdilution method and a time-kill assay. (**a**) A representative image of a microdilution plate after cultivation for 2 days. Red lines indicate the border separating wells with and without a button of ≥ 1 mm. (**b**) Colony-forming unit (CFU) counting of cultures with and without INH at 16 µg mL^−1^ resulting from broth microdilution assays. The bars represent the mean CFU mL^−1^ values of 12 (MC^2^155, Δ*pzaA*, and MC^2^155 pMV261-*pzaA*) or six (MC^2^155 pMV261) replicate cultures originating from different colonies. The error bars represent the standard deviations. *p < 0.01 versus MC^2^155 (Mann–Whitney *U* test). (**c**) Time-kill curves of INH (64 µg mL^−1^) against MC^2^155 and Δ*pzaA* strains cultivated in nitrogen-depleted M9. Data are expressed as fold-change of the number of CFUs relative to 0-h samples. Dots and lines indicate individual values and the mean of triplicate cultures.

To determine whether the production of PzaA can promote the INH resistance of *M. smegmatis*, we constructed a strain constitutively expressing PzaA by introducing the pMV261-*pzaA* plasmid into MC^2^155. When we grew this strain in 7H9 broth for 40 h and performed an enzyme assay with cell-free extracts, the isonicotinic acid-forming activity was determined to be 0.0201 μmol min^−1^ mg^−1^, which was considerably higher than the activity of the parental MC^2^155 cells grown in 7H9 (0.000717 [SD 0.0000192] μmol min^−1^ mg^−1^) and about half the level of MC^2^155 grown under the optimized conditions (0.0435 [SD 0.000833] μmol min^−1^ mg^−1^), demonstrating that this strain constantly produces PzaA. Then, we subjected this strain, together with MC^2^155, Δ*pzaA*, and an empty vector control (MC^2^155 pMV261), to antimicrobial susceptibility tests with INH by the microdilution method using 7H9 broth as the growth media. The minimum inhibitory concentration (MIC) of INH against MC^2^155 pMV261-*pzaA* was determined to be 32 µg mL^−1^ whereas that against the other strains was 16 µg mL^−1^, suggesting that PzaA expressed in cells can increase the resistance of *M. smegmatis* to INH (Fig. 5a). As the observed difference in the growth levels between MC^2^155 pMV261-*pzaA* and the other three was subtle, we also performed colony-forming unit (CFU) counting for some of the cultures in the wells of the microdilution plates (the ones with and without INH at 16 µg mL^−1^) (Fig. 5b). Consistent with the MIC readings, the determined CFUs of MC^2^155 pMV261-*pzaA* cultures were approximately fourfold higher than those of the cultures of the other strains after incubation with INH (16 µg mL^−1^). However, our data must be interpreted with caution, as no difference was observed between the growth of MC^2^155 and that of Δ*pzaA*. We reasoned that the lack of difference between these strains may be attributed to the lack of PzaA expression in 7H9 broth. To confirm this idea, we measured the transcription levels of *pzaA* in MC^2^155 after cultivation in 7H9 and nitrogen-depleted M9 for 6 h in the absence and presence of INH (0.5 mM). As expected, a strong induction of *pzaA* transcription by INH occurred in nitrogen-depleted M9 but not in 7H9 broth (Supplementary Fig. S2).

To further analyze the relationship between the production of PzaA and INH resistance, we performed a time-kill assay for MC^2^155, ∆*pzaA*, ∆*pzaA* pMV261-*pzaA*, and ∆*pzaA* pMV261. In this experiment, we cultivated these strains in nitrogen-depleted M9 with or without INH (64 µg mL^−1^) for 72 h, periodically sampling the cultures and counting CFU (Fig. 5c). As for the cultures without added INH, the CFUs for all the strains gradually declined over time even in the absence of an antimicrobial. As expected, no growth was observed due to the lack of a nitrogen source in the medium. However, contrary to our expectation, MC^2^155 did not only survive the exposure to INH at 64 µg mL^−1^ but also began to grow at around 12 h of cultivation in the presence of INH. In contrast, the other three strains did not show such a growth in the presence of INH, although they were similar to MC^2^155 in that they exhibited little or no sensitivity to the killing effect of INH. These observations suggested the possibility that MC^2^155 was able to grow in the presence of INH because it could utilize INH as a nitrogen source via hydrolysis of this compound by PzaA. We then aimed to determine the reason why ∆*pzaA* pMV261-*pzaA* could not grow on INH as a nitrogen source like MC^2^155 did, even though it had an intact *pzaA* gene on a plasmid. Therefore, we performed a whole-cell enzyme assay using cells of MC^2^155 and ∆*pzaA* pMV261-*pzaA* grown in nitrogen-depleted M9 supplemented with INH (64 µg mL^−1^) for 24 h, suspecting that this *pzaA*-complemented strain might not be expressing PzaA in the cells as highly as the parental strain under the given growth conditions. Indeed, the INH-hydrolyzing activity of the *pzaA*-complemented strain was determined to be 7.67 (SD 1.74) μM h^−1^ OD^−1^, less than one-seventh the activity of MC^2^155 (58.6 [SD 8.93] μM h^−1^ OD^−1^). Collectively, we concluded that PzaA has a positive effect on the survival and growth of *M. smegmatis* in the presence of INH, presumably as a resistance factor and a tool that enables the use of INH as a nutrient.

### Exposure to INH promotes the emergence of an INH-resistant mutant that can express PzaA at high levels under nitrogen-rich conditions

If PzaA functions as an intrinsic INH resistance factor, mutants that highly express PzaA even in nitrogen-rich media may emerge in circumstances where INH exists but PzaA is not expressed due to the presence of nitrogen sources. Therefore, we performed a screening for spontaneous INH-resistant mutants by spreading a culture of MC^2^155 on 7H11 agar plates containing INH (100 µg mL^−1^) and obtaining colonies formed on the plates after cultivation for 5 days. Screening of approximately 3 × 10^8^ cells yielded 408 INH-resistant mutants, from which we randomly selected 30 for further analysis. Notably, in a whole-cell enzyme assay using cells grown in 7H9 medium containing INH (100 µg mL^−1^) for 40 h, one of these mutants showed an INH-hydrolyzing activity at 70.1 (SD 10.7) μM h^−1^ OD^−1^ (measured by monitoring the formation of isonicotinic acid), a rate even higher than that of MC^2^155 cells cultivated under optimized PzaA-inducing conditions (64.2 [SD 11.9] μM h^−1^ OD^−1^). Activities of the other 29 strains were within a normal range (0.772–2.22 µM h^−1^ OD^−1^; measured by monitoring the formation of hydrazine).

To confirm that the increased hydrazidase activity of the mutant resulted from the increased production of PzaA, we measured the transcription level of *pzaA* in the mutant cells after cultivation in a 7H9 medium containing INH (100 µg mL^−1^) for 40 h. The *pzaA* mRNA level (measured in triplicate) was 114 (SD 27.2)-fold higher than the level of MC^2^155 grown in 7H9 medium without INH. We believe these observations support the view that this strain has a modified gene regulation system that allows the expression of PzaA in nitrogen-rich media and that the production of PzaA confers a growth advantage to the mutant in the presence of INH.

### Distribution of PzaA homologs in mycobacteria

**Figure 6:**
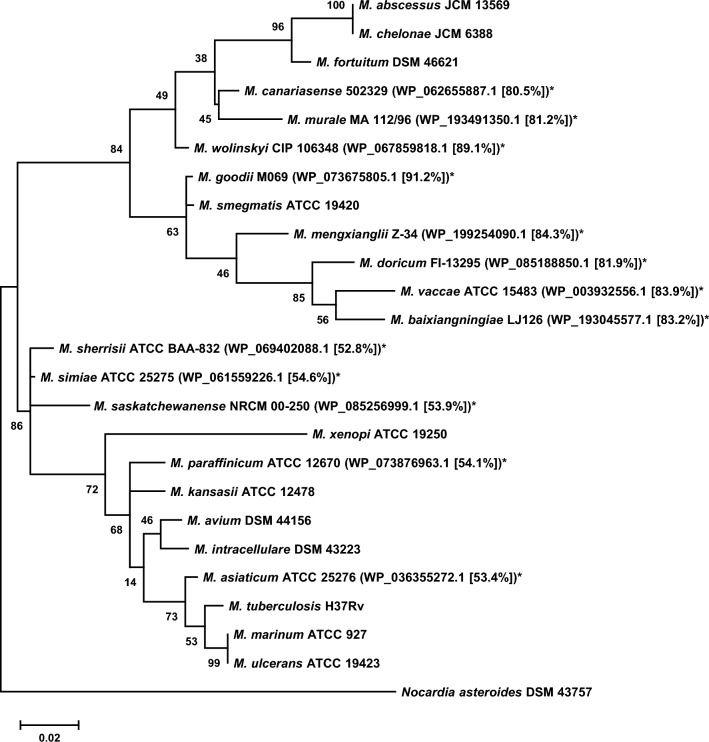
Phylogenetic tree of mycobacteria generated using 16S rRNA gene sequences. Bootstrap values are shown at each node. Asterisks indicate species that have a gene orthologous to *pzaA*. NCBI accession numbers of the PzaA-like proteins and the percent identity of their amino acid sequences with *Mycobacterium smegmatis* PzaA are shown in parentheses. The bar represents 0.02 substitutions per site.

To investigate whether and how *pzaA* is distributed among mycobacteria, we searched the NCBI non-redundant protein sequence (nr) database, limiting the target to *Mycobacteriaceae* (taxonomy id: 1762), for homologs of PzaA using BLASTP algorithm. We found 34 entries, excluding PzaA itself, that satisfied the criteria of query coverage and percent identity being ≥ 80% and ≥ 50%, respectively (Supplementary Table S1). These *pzaA*-like sequences were from 13 described and 8 undescribed mycobacterial species. To determine the distribution pattern of the *pzaA*-like genes among mycobacteria, we performed a phylogenetic analysis using 16S rRNA gene sequences of these 13 described species, together with sequences of some important human pathogens, including Mtb, *M. avium*, *M. intracellulare*, *M. abscessus*, and *M. kansasii* (Fig. 6). The strains were divided into two major clades, one containing slow-growing species and the other containing rapid-growing ones, and the *pzaA*-like gene possessors were identified in both groups. The amino acid sequence identity of the PzaA-like gene products to *M. smegmatis* PzaA generally agreed well with the phylogenetic distance of each species possessing the genes from *M. smegmatis*, suggesting that these genes were orthologous to each other.

To confirm if the pathogens that seemed to lack *pzaA* actually lack this gene, or if we missed candidate genes because the cut-off value of percent identity that we used (50%) was too high, we searched for *pzaA* homologs in mycobacteria targeting those with < 50% amino acid sequence identity. All the human pathogenic strains, for which we checked the genome, were found to have three to six PzaA homologs that satisfied the criteria of query coverage and percent identity being ≥ 80% and ≥ 30%, respectively, and were annotated as Asp-tRNA^Asn^/Glu-tRNA^Gln^ amidotransferase subunit GatA or putative amidase. However, the amino acid sequence identities of these homologs to PzaA, which fell within the range of 30–35%, were too low to be considered orthologs of PzaA. In addition, some of those homologs were more similar to paralogs of *pzaA* identified in the *M. smegmatis* genome than to *pzaA* itself. These findings support our view that some human pathogenic mycobacteria lack genes orthologous to *pzaA*.

## Discussion

Hydrazidase is a type of enzyme that hydrolyzes hydrazides, chemicals that can be formed by a condensation reaction between hydrazine (H_2_N-NH_2_) and carbonic acid, sulfonic acid, or phosphoric acid^12^. INH is the most well-known and useful hydrazide indispensable to combat infectious diseases caused by mycobacteria. Several studies published from the 1950s to 1970s demonstrated that some mycobacteria, including *M. avium* and *M. smegmatis*, produce an INH-hydrolyzing enzyme^7,8,10^. Although it was not clear if hydrazidase was involved, other studies also demonstrated the production of an INH-degrading or -inactivating agent, presumably an enzyme, by Mtb and *M. bovis* BCG^13,14^. However, to date, no study has revealed the identity of these INH-degrading enzymes. To the best of our knowledge, the present study is the first to reveal the identity of mycobacterial hydrazidase. PzaA, the herein-identified enzyme, had been known as nicotinamidase/pyrazinamidase with unclear physiological roles. Our data showed that it degrades nicotinamide and pyrazinamide considerably more efficiently than it degrades INH. Nevertheless, we believe that it may still function as “hydrazidase” rather than “nicotinamidase/pyrazinamidase” in natural settings because, among the chemicals we tested, only INH served as an efficient inducer of gene expression and because our INH susceptibility tests demonstrated that its expression at high levels is beneficial to the survival of *M. smegmatis* in the presence of INH. In addition, MC^2^155, but not ∆*pzaA*, showed growth in nitrogen-depleted M9 when the medium was supplemented with INH, indicating that MC^2^155 was using INH as a nitrogen source and that PzaA was necessary for this activity. Furthermore, we successfully showed that a spontaneous INH-resistant mutant that highly expresses PzaA even in nitrogen-rich media emerges under the selective pressure of INH imposed in 7H9, suggesting that the production of this enzyme confers a growth advantage on the bacterium in the presence of INH. Overall, these results suggest that PzaA may have a role as an intrinsic INH resistance factor and/or a tool to use INH as a nutrient.

In addition, our BLASTP search and phylogenetic analysis of PzaA homologs indicated that some important pathogenic mycobacteria, including *M. avium*, Mtb, and *M. bovis* BCG, lack a protein orthologous to PzaA. This leads us to speculate about the enzymes reported by Toida^7,8^, Youatt^14^, and Youmans and Youmans^13^ in some mycobacterial strains that are supposed to lack PzaA. We suspect that some amidases other than PzaA may be responsible for the degradation of INH in those mycobacteria, especially in *M. avium*, in which the formation of hydrazine was observed. Youatt has suggested that there are two enzyme systems in *M. bovis* BCG, one forming isonicotinic acid and the other forming both isonicotinic acid and 4-pyridyl methanol via the formation of 4-pyridine aldehyde^14,15^. In addition to hydrazidase, *N*-acetyltransferases may serve as a candidate for the former enzyme that produced isonicotinic acid because studies published later by other groups demonstrated that Mtb and *M. bovis* BCG have *N*-acetyltransferases that catalyze the acetylation of INH, following which, according to Arun et al., acetylisoniazid is somehow hydrolyzed into isonicotinic acid and acetylhydrazine^16–18^. Notably, hydrazidase might also be involved in the hydrolysis of this acetylisoniazid. Therefore, we believe that it is worth trying to purify and identify INH-hydrolyzing enzymes from human pathogens to obtain a better understanding of the distribution and clinical impact of mycobacterial hydrazidase.

This study had several limitations. First, although we have shown several different data sets that support the notion that the production of PzaA is beneficial to the survival and growth of *M. smegmatis* in the presence of INH, each set has some weaknesses. For example, our INH susceptibility test by the broth microdilution method failed to demonstrate the benefit of PzaA expressed from the natural promoter. In the time-kill assay, we did not successfully complement the growth defect of ∆*pzaA* in nitrogen-depleted M9 containing INH with a PzaA-expressing plasmid, presumably because the PzaA expression level was not sufficient. Additionally, although we showed that an INH-resistant mutant that highly expresses PzaA in nitrogen-rich media emerges under the selective pressure of INH, we did not reveal the molecular mechanism behind this phenomenon. Therefore, our data should be cautiously interpreted and these phenomena should be further investigated in future studies. Second, the mechanism behind the INH-induced high expression of PzaA remains unclear. We have recently discovered that a LuxR-type regulator, coded by a gene (MSMEI_1050) located in the same gene cluster as *pzaA*, is involved in the INH-dependent transcriptional regulation of PzaA^19^. The gene deletion of MSMEI_1050 deprived *M. smegmatis* of the ability to respond to INH, and complementation with an MSMEI_1050 expression plasmid restored this ability. In addition, we have found that the transcription of MSMEI_1050 was inhibited by the presence of NH_4_Cl (0.1%). These findings suggest that the lack of expression of PzaA under nitrogen-rich conditions can be attributed to the lack of this transcriptional regulator. However, we have not yet obtained biochemical data on the regulator, and whether it directly responds to INH is unknown. We believe that our attempt to biochemically characterize the regulator may clarify the INH-dependent induction mechanism of PzaA. Lastly, perhaps the most important question “Can INH hydrolysis be regarded as one of the physiological roles of PzaA?” remains to be addressed. Before answering this question, we may need to determine whether there are natural circumstances where the high expression of PzaA in response to exposure to INH is beneficial to *M. smegmatis*. There are many natural settings where nitrogen source is limited. However, in such natural settings, *M. smegmatis* is unlikely to be occasionally exposed to INH, especially because INH has not been known as a natural product. Perhaps, the most simple and likely interpretation of our findings is that PzaA exists to play a role in cellular nicotinamide or related amide metabolism and its INH-induced expression and INH hydrolysis were artificial observations that do not naturally occur. As an alternative theory, we speculate that INH may also exist as a natural product from some bacteria, fungi, or plants, and some mycobacteria might have developed a defense system against this toxic compound.

## Methods

### Bacterial strains, plasmids, and culture conditions

*M. smegmatis* strains were routinely cultured at 37 °C in Middlebrook 7H9 broth (Becton–Dickinson, Franklin Lakes, NJ, USA) supplemented with albumin-dextrose-catalase, 0.2% glycerol, and 0.05% Tween 80 with shaking at 130 rpm, or on Middlebrook 7H11 agar (Becton–Dickinson) plates with oleic acid-albumin-dextrose-catalase enrichment and 0.5% glycerol. Unless otherwise stated, 7H9 and 7H11 media contained the abovementioned supplements in any other experiments. *Escherichia coli* DH5α, which was used for plasmid manipulation, was cultured in Luria–Bertani broth or agar plates.

### Enzyme assays with resting cells or protein samples

The INH-degrading activity of *M. smegmatis* cells grown under different conditions was determined as follows: cells collected by centrifugation (7500 × *g*, 10 min) from 10 mL of the cultures were washed twice with 50 mM sodium phosphate buffer (pH 7.0) (buffer A) and resuspended in one-tenth of the original buffer volume. Next, 450 μL of the cell suspension was transferred into 2-mL tubes, mixed with 50 μL of 100 mM INH solution, and incubated at 37 °C with shaking at 110 rpm for 3 h. The cells were removed by centrifugation (21,000 × *g*, 2 min, 4 °C), and the resultant supernatant was used for the measurement of either isonicotinic acid or hydrazine. For isonicotinic acid measurement, 100 μL of the supernatant was mixed with 400 μL of acetonitrile, filtered with a 4-mm Millex GV 0.22-μm syringe filter (Merck Millipore, Darmstadt, Germany), and analyzed with an ACQUITY UPLC H-Class system (Waters, Milford, MA, USA) equipped with a PDA eλ detector and a BEH amid column (2.1 × 100 mm) (Waters). The isocratic mobile phase was a mixture of acetonitrile and 10 mM ammonium acetate (pH 9.0) in a 9:1 (v/v) ratio, pumped at a flow rate of 0.8 mL min^−1^. The temperature in the column oven and autosampler was set to 50 °C and 10 °C, respectively. The injection volume and wavelength for INH and isonicotinic acid detection were 2 μL and 260 nm, respectively. To measure hydrazine, 400 μL of the supernatant was mixed with 100 μL of 25% trichloroacetic acid, centrifuged at 21,000 × *g* for 5 min, and then subjected to a colorimetric assay using *p*-dimethylaminobenzaldehyde, with which hydrazine reacts to form a yellow compound with strong absorbance at 458 nm, as described elsewhere^7^. A standard curve was generated and used to determine the concentration of hydrazine.

The INH-degrading activity of the crude or purified enzyme was measured by a colorimetric method that monitors hydrazine formation and a UPLC-based method that detects isonicotinic acid. In either method, the reaction mixtures comprised 10 mM INH and an appropriate amount of enzyme in buffer A in a total volume of 200 μL. Unless otherwise stated, the reaction was performed at 37 °C for 15 min for purified and 3 h for crude samples and terminated by adding 800 μL of acetonitrile for detecting isonicotinic acid or 50 μL of 25% trichloroacetic acid for detecting hydrazine. Each product was analyzed in the same manner as in the assays with resting cells.

In steady-state kinetics experiments, INH- and nicotinic acid hydrazide-hydrolyzing activities were evaluated using the colorimetric method that monitors the formation of hydrazine as described above, except that the reaction mixtures contained varying concentrations (0.313–20 mM) of INH or nicotinic acid hydrazide as a substrate. The amide-hydrolyzing activity of purified PzaA was measured by a colorimetric method that monitored ammonia formation as previously described^20^. The reaction mixtures comprised isonicotinamide, nicotinamide, or pyrazinamide (0.156–5 mM) as substrates and an appropriate amount of enzyme in buffer A in a total volume of 200 μL. The reaction was carried out at 37 °C for 15 min and terminated by the addition of 50 μL of 200 mM sodium hydroxide solution.

### Purification and identification of the INH-degrading enzyme

*M. smegmatis* MC^2^155 cells cultured in 2 L of 7H9 medium were harvested by centrifugation (10,000 × *g*, 30 min), resuspended in 2 L of nitrogen-depleted M9 supplemented with INH (0.5 mM), and cultured at 37 °C for an additional 6 h with shaking at 110 rpm. Cells were harvested by centrifugation (10,000 × *g*, 10 min, 4 °C), washed twice with buffer A, and resuspended in 20 mL of buffer A. Cells were lysed by sonication, and the insoluble material was removed by centrifugation (6000 × *g*, 30 min, 4 °C). All purification steps were performed at 0–4 °C. The INH-degrading enzyme was first fractionated in a 40–60% saturated ammonium sulfate cut. After redissolving in 12 mL of buffer A and desalting through dialysis, the fraction was applied to a HiTrap Q HP 5 mL column (GE Healthcare, Chicago, IL, USA) equilibrated with buffer A. The column was washed with 10 mL of buffer A, and bound proteins were eluted with a 50 mL linear gradient of 0–0.5 M NaCl. The active fractions were collected and mixed with an appropriate volume of 80% saturated ammonium sulfate solution to achieve 20% saturation. The resulting mixture was applied to a HiTrap Butyl HP 5 mL column (GE Healthcare) equilibrated with 50 mL of buffer A supplemented with 20% saturated ammonium sulfate. The column was washed with 10 mL of the same buffer, and bound proteins were eluted in a 50 mL linear gradient of 20–0% saturated ammonium sulfate. The active fractions were pooled, concentrated from 5 to 0.25 mL using an Amicon Ultra-15 (3,000 kDa MWCO), and applied to a Superdex 200 Increase 10/300 GL (GE Healthcare) equilibrated with buffer A supplemented with 150 mM NaCl. Proteins were eluted in the same buffer, and the active fractions were collected. The resultant sample was applied to a Resource Q 1 mL column (GE Healthcare) equilibrated with buffer A. The column was washed with 2 mL of buffer A, and the bound proteins were eluted with a 20 mL linear gradient of 0–0.5 M NaCl. Active fractions were collected and subjected to purity check by SDS-PAGE and protein concentration measurement using a Bio-Rad Protein Assay Dye Reagent Concentrate (Bio-Rad, Hercules, CA, USA) with BSA as the standard.

The purified protein was identified using peptide mass fingerprinting. After SDS-PAGE of the purified material, the major band was excised from the gel and digested with trypsin. Matrix-assisted laser desorption/ionization-time of flight-mass analysis with Microflex LRF 20 (Bruker Daltonics, Bremen, Germany) was performed using a saturated solution of α-cyano-4-hydroxycinnamic acid in 50% acetonitrile containing 0.1% trifluoroacetic acid as the matrix, as previously described^21^. A peak list was generated using Flex Analysis 3.0 software (Bruker Daltonics) and analyzed using the MASCOT program (Matrix Science Ltd., London, UK) with the following parameters: trypsin as the cleaving enzyme, a maximum of one missed cleavage, iodoacetamide (Cys) for complete modification, oxidation (Met) for partial modification, monoisotopic masses, and a mass tolerance of ± 0.1 Da.

### Expression and analysis of PncA

The PncA-expressing strain (Δ*pzaA* pMyC-*pncA*) was constructed as follows. pMyC, an acetamide-inducible expression plasmid, was a gift from Annabel Parret & Matthias Wilmanns (Addgene plasmid #42192; http://n2t.net/addgene:42192; RRID: Addgene_42192)^22^. The *pncA* structural gene was amplified by PCR using the primers Exp_PncA_F and Exp_PncA_R (Supplementary Table S2) and fused to NcoI- and HindIII-digested pMyC using In-Fusion HD cloning kit (Takara Bio, Shiga, Japan). The resulting plasmid pMyC-*pncA* was electroporated into ∆*pzaA*. Transformants were selected using 7H11 agar plates containing hygromycin (100 µg mL^−1^).

To express PncA, ∆*pzaA* pMyC-*pncA* was grown to an OD_600_ of 1 in 7H9 media containing hygromycin (100 µg mL^−1^) and then induced with acetamide (final 0.2%) for 24 h. The cultured cells were washed twice with buffer A and resuspended in one-tenth of the original buffer volume. Cells were lysed by sonication, and the insoluble material was removed by centrifugation at 140,000 × *g* for 30 min at 4 °C. The amide- and INH-hydrolyzing activities of cell-free extracts were measured by the colorimetric methods that monitored the formation of hydrazine or ammonia as described in the section titled “Enzyme assays with resting cells or protein samples”.

### Quantitative reverse transcription PCR (qRT-PCR)

qRT-PCR was performed to assess the ability of INH and other chemicals to induce *pzaA* transcription. *M. smegmatis* MC^2^155 cells cultured in 50 mL of 7H9 medium at 37 °C for 40 h were harvested by centrifugation (7500 × *g*, 10 min) and suspended in 50 mL of M9-based liquid medium (or in 7H9 broth when testing *pzaA* transcriptional induction by INH in this medium) with or without a chemical for testing (INH, nicotinic acid hydrazide, isonicotinamide, nicotinamide, or pyrazinamide; 0.5 mM each). The suspensions were cultured at 37 °C, and at the indicated time points, 2 mL of each suspension was collected for use in qRT-PCR analysis.

Total RNA was extracted using the hot phenol method, and cDNA was synthesized using a PrimeScript RT Reagent Kit with gDNA Eraser (Takara Bio)^23^. cDNA was evaluated by qRT-PCR using an Applied Biosystems PowerUp SYBR Green Master Mix (Thermo Fisher Scientific, Waltham, MA, USA) in an Applied Biosystems 7500 Fast Real-Time PCR System (Thermo Fisher Scientific). The transcript levels of each gene were calculated using the 2^−ΔΔCt^ method^24^. Primers used for qRT-PCR are listed in Supplementary Table S2. mRNA expression of *pzaA* was normalized against that of *sigA*, and the resulting data were used to calculate fold changes in the *pzaA* transcript levels before and after incubation with an inducer chemical.

### Construction of *pzaA* deletion mutant and constitutively expressing strain

Electrotransformation of *M. smegmatis* was performed using a Gene Pulser Xcell (Bio-Rad) as previously described^25^. Nucleotides were sequenced by the dideoxy chain termination method using a 3130xl automated DNA sequencer (Thermo Fisher Scientific).

*M. smegmatis* MC^2^155 Δ*pzaA* was constructed by a two-step allelic exchange with the suicide vector pK18*mobsacB*^26^ (provided by the National Institute of Genetics through the National BioResource Project of the MEXT, Japan). A mutant allele of *pzaA* with an in-frame deletion was constructed by splicing via overlap extension PCR^27^ using primers Dis_pzaA_1, Dis_pzaA_2, Dis_pzaA_3, and Dis_pzaA_4 (Supplementary Table S2) and inserted into the multi-cloning site of pK18*mobsacB*. This resulting plasmid was introduced into *M. smegmatis* MC^2^155 by electrotransformation, following which the cells were spread on 7H11 agar plates containing kanamycin (20 µg mL^−1^) and cultivated at 37 °C for 3–5 days. After cultivations, strains that formed colonies were selected as first-crossover mutants that contained the whole plasmid integrated into the chromosome. To obtain mutants that lost the plasmid through a second-crossover event, these first-crossover mutants were cultivated in 7H9 broth for 48 h at 37 °C with shaking at 130 rpm and spread on 7H11 agar plates containing 10% sucrose. Finally, mutants with Δ*pzaA* were selected based on the size of the PCR amplicon generated using primers Dis_pzaA_1 and Dis_pzaA_4 and further confirmed by Sanger sequencing of the amplicon.

The strain constantly expressing PzaA (MC^2^155 pMV261-*pzaA*) was constructed as follows. The *pzaA* structural gene was amplified by PCR using the primers Exp_PzaA_F and Exp_PzaA_R (Supplementary Table S2) and inserted into the multi-cloning site of pMV261^28^ using the PstI and HindIII restriction sites. The resulting expression vector, pMV261-*pzaA*, was electroporated into *M. smegmatis* MC^2^155, which yielded the strain of interest. Kanamycin (20 µg mL^−1^) was used for the selection and routine growth of this strain and MC^2^155 pMV261, which we prepared to use as an empty vector control strain.

### Antimicrobial susceptibility testing

We used the broth microdilution method for determining the MIC of INH against *M. smegmatis* strains. INH at concentrations ranging from 128 to 0.5 µg mL^-1^ was prepared by twofold serial dilution using 7H9 broth and added to wells of 96-well round-bottom plates at 150 μL per well. The tested strains, pre-grown in 7H9 at 37 °C for 2 days, were added to the wells to provide a final inoculum density of approximately 5 × 10^4^ CFUs mL^−1^. After 2 days of incubation at 37 °C, the MICs were determined as the lowest concentrations of INH that significantly prevented the growth of the tested strains (i.e. the lowest concentration at which a button of < 1 mm was observed). Additionally, some of the cultures in the wells (the ones that contained INH at 16 µg mL^−1^ and that did not contain INH) were subjected to CFU counting as follows: ten-fold serial dilutions of the cell suspensions were plated on 7H11 agar plates. After incubation for 3 days at 37 °C, colonies were counted, and the number of CFUs was calculated.

### Time-kill assay

The time-kill assay of INH against MC^2^155, Δ*pzaA*, Δ*pzaA* pMV261-*pzaA*, and Δ*pzaA* pMV261 was performed as follows. The tested strains were pre-grown in 7H9 media with kanamycin at 20 µg mL^−1^ (for Δ*pzaA* pMV261-*pzaA* and Δ*pzaA* pMV261) or without an antimicrobial (for MC^2^155 and Δ*pzaA*) at 37 °C for 40 h. They were then inoculated into nitrogen-depleted M9 with or without INH (64 µg mL^−1^) at a final cell density of 1 × 10^5^ CFUs mL^−1^ and cultivated at 37 °C for 72 h without shaking. At several time points during cultivation, a small portion was removed from each culture, serially diluted tenfold, and plated on 7H11 agar plates. After incubation for 3 days at 37 °C, colonies formed on the plates were counted to determine the number of CFUs per milliliter of the original cultures.

### Isolation and analysis of spontaneous INH-resistant mutants

MC^2^155 were grown in 7H9 medium for 24 h at 37 °C, and then a portion was diluted to an OD_600_ of 1.0 and spread on 7H11 agar plates containing INH (100 µg mL^-1^). After cultivation for 5 days at 37 °C, strains that grew on the plates were selected as spontaneous INH-resistant mutants and subjected to a whole-cell INH-hydrolyzing enzyme assay that monitored hydrazine formation. One strain that showed a heightened INH-hydrolyzing activity was further subjected to a whole-cell INH-hydrolyzing enzyme assay that monitored isonicotinic acid formation and qRT-PCR to test the *pzaA* mRNA level. In all these assays, the tested strains were inoculated in a 7H9 medium containing INH (100 µg mL^−1^) to a starting OD_600_ of 0.05 and cultivated at 37 °C for 40 h, before harvesting by centrifugation. The inoculum was from a 40-h culture grown in 7H9 containing INH (100 µg mL^−1^). Whole-cell enzyme assays and qRT-PCR were performed as described above in the sections titled “Enzyme assays with resting cells or protein samples” and “Quantitative reverse transcription PCR (qRT-PCR)”.

### Phylogenetic analysis

All 25 bacterial strains included in the phylogenetic analysis were the type strains of each species. The strain identities and GenBank accession numbers of the 16S rRNA gene sequences are as follows (the latter is shown in parentheses): *M. smegmatis* ATCC 19420 (AJ131761), *M. abscessus* JCM 13569 (AB548599), *M. asiaticum* ATCC 25276 (X55604), *M. avium* DSM 44156 (AJ536037), *M. canariasense* 502329 (AY255478), *M. chelonae* JCM 6388 (AB548610), *M. doricum* FI-13295 (AF264700), *M. fortuitum* DSM 46621 (AJ536039), *M. goodii* M069 (Y12872), *M. intracellulare* DSM 43223 (AJ536036), *M. kansasii* ATCC12478 (KY934282), *M. marinum* ATCC 927 (AF456240), *M. murale* MA 112/96 (Y08857), *M. paraffinicum* ATCC 12670 (X88925), *M. saskatchewanense* NRCM 00-250 (AY208856), *M. sherrisii* ATCC BAA-832 (AY353699), *M. simiae* ATCC 25275 (X52931), *M. tuberculosis* H37Rv (MH794239), *M. ulcerans* ATCC 19423 (AB548725), *M. wolinskyi* CIP 106348 (AF547981), *M. xenopi* ATCC 19250 (MH169221), *M. baixiangningiae* LJ126 (MT466538), *M. mengxianglii* Z-34 (MN861241), *M. vaccae* ATCC 15483 (X55601), and *Nocardia asteroides* DSM 43757 (AF430019). A phylogenetic tree was constructed by the maximum likelihood method and Kimura 2-parameter model^29^ using the MEGA X program^30^. The number of bootstrap replications was set to 1000.

### Statistical analysis

The Mann–Whitney *U* test was performed using R v.4.1.2 (The R Foundation for Statistical Computing, Vienna, Austria). The tests were used to analyze data presented in Fig. 5b. A *p*-value < 0.05 indicated that the difference in values between the two groups was statistically significant. Means and SDs of enzyme activities were calculated from triplicate measurements.

### Equipment and settings

The SDS-PAGE gel image shown in Fig. 3 was taken using a Limited-Stage II gel imager (AMZ System Science, Osaka, Japan), equipped with an EOS Kiss X7i camera (Canon, Tokyo, Japan) with an EF-S18-55 mm lens, and a standard white-light box, on which the gel was placed. Aperture, shutter speed, and ISO were set to F20, 1/6 s, and 100, respectively. No image processing was performed.

## Supplementary Information

Supplementary Information.

## Data Availability

All data generated or analyzed during this study are included in this published article. Requests for more details should be addressed to the corresponding author.
