# Supplementary material for: Identification of a mycobacterial hydrazidase, an isoniazid-hydrolyzing enzyme
Source: Sci Rep. 2023 May 20;13:8180. doi: 10.1038/s41598-023-35213-5 (PMC10199920; doi:10.1038/s41598-023-35213-5)
Supplement: Supplementary file 1 — Supplementary Information. [file 41598_2023_35213_MOESM1_ESM.pdf]

Supplementary Information for

Identification of a mycobacterial hydrazidase, an isoniazid-hydrolyzing enzyme

Arata Sakiyama[1], Chaogetu Saren[1], Yukihiro Kaneko[2,3], and Ken-Ichi Oinuma[2,3,*]

[1]Department of Bacteriology, Osaka City University Graduate School of Medicine, Abeno-ku, Osaka, Japan

[2]Department of Bacteriology, Osaka Metropolitan University Graduate School of Medicine, Abeno-ku, Osaka, Japan

[3]Research Center for Infectious Disease Sciences, Osaka Metropolitan University Graduate School of Medicine, Abeno-ku, Osaka, Japan

* Address correspondence to Ken-Ichi Oinuma, kioinuma@omu.ac.jp.

Tel.: +81 6-6645-3746; Fax: +81 6-6645-3747

Supplementary Data S1. MASCOT search results.

Protein View: WP_011727401.1

Asp-tRNA(Asn)/Glu-tRNA(Gln) amidotransferase subunit GatA [Mycobacterium smegmatis]

**Database:**	NCBIprot
**Score:**	358
**Expect:**	3.3e-29
**Monoisotopic mass (M_r):**	49163
**Calculated pI:**	4.79
**Taxonomy:**	Mycolicibacterium smegmatis

This protein sequence matches the following other entries:

- YP_885485.1 from Mycolicibacterium smegmatis MC2 155
- ABK71083.1 from Mycolicibacterium smegmatis MC2 155
- AFP37538.1 from Mycolicibacterium smegmatis MC2 155
- AIU06340.1 from Mycolicibacterium smegmatis MC2 155
- AIU12965.1 from Mycolicibacterium smegmatis
- AIU19589.1 from Mycolicibacterium smegmatis

Sequence similarity is available as an NCBI BLAST search of WP_011727401.1 against nr.

Search parameters

**Enzyme:**	Trypsin: cuts C-term side of KR unless next residue is P.
**Variable modifications:**	Oxidation (M)
**Mass values searched:**	54
**Mass values matched:**	35

Protein sequence coverage: 64%

Matched peptides shown in ***bold red***.

```
  1 MELYELPLIE VAEKIRTKEV SPVEVAESSL ARLEEVEPLL TAFVTTTPEL
 51 ALEQAKAAEK EIADGKYRGP LHGIPLGVKD LYDTAGIRTT SSSAQRADYV
101 PDADSVSVAK LYDAGMVLVG KTHTHEFAYG ATTPTTGNPW APDRTPGGSS
151 GGSGAAVAAG VVHVALGSDT GGSIRIPAAL CGTVGLKPTY GRASRVGVAS
201 LSWSLDHVGP LSRNVTDAAL VMQAMSGYDR RDPGTANVAV PDMVSGIDAG
251 VAGKKIGIPV NYYTDRVAPE AAEAAKVAAA TFEKLGAQLV EVEIPMAEHI
301 VPTEWAIMMP EATAYHMDYL RNSPEKFTDE VRTLLEVGAA EPAVDYVNAM
351 RLRTLIQAAW NEMFTGIDVL LAPTVVAPAT LRSDPFVRWE DGTVEAATAA
401 YVRLSAPANV TGLPSLSVPA AFTADGLPLG VQILGKPFAE PEILTFGYAL
451 EQNTDTVGRI APVLEKVG
```

Unformatted sequence string: 468 residues (for pasting into other applications).

Sort by (●) residue number ( ) increasing mass ( ) decreasing mass
Show (●) matched peptides only ( ) predicted peptides also

Start – End	Observed	Mr(expt)	Mr(calc)	Delta	M	Peptide
1 – 14	1676.8949	1675.8876	1675.8742	0.0134	0	-.MELYELPLIEVAEK.I
1 – 14	1692.8945	1691.8872	1691.8691	0.0181	0	-.MELYELPLIEVAEK.I + Oxidation (M)
17 – 32	1701.9222	1700.9149	1700.8945	0.0205	1	R.TKEVSPVEVAESSLAR.L
19 – 32	1472.7434	1471.7361	1471.7518	-0.0157	0	K.EVSPVEVAESSLAR.L
33 – 56	2642.5653	2641.5581	2641.4102	0.1479	0	R.LEEVEPLLTAFVTTTPELALEQAK.A
61 – 68	951.3684	950.3611	950.4821	-0.1210	1	K.EIADGKYR.G
67 – 79	1406.7989	1405.7916	1405.8194	-0.0278	1	K.YRGPLHGIPLGVK.D

Start – End	Observed	Mr(expt)	Mr(calc)	Delta	M	Peptide
69 – 79	1087.5834	1086.5761	1086.6550	-0.0788	0	R.GPLHGIPLGVK.D
69 – 88	2092.1715	2091.1643	2091.1477	0.0166	1	R.GPLHGIPLGVKDLYDTAGIR.T
80 – 88	1023.4148	1022.4075	1022.5033	-0.0958	0	K.DLYDTAGIR.T
80 – 96	1841.8866	1840.8793	1840.8915	-0.0122	1	K.DLYDTAGIRTTSSSAQR.A
111 – 121	1165.5711	1164.5638	1164.6213	-0.0575	0	K.LYDAGMVLVGK.T
111 – 121	1181.5662	1180.5589	1180.6162	-0.0573	0	K.LYDAGMVLVGK.T + Oxidation (M)
122 – 144	2528.2440	2527.2367	2527.1517	0.0851	0	K.THTHEFAYGATTPTTGNPWAPDR.T
145 – 175	2652.4660	2651.4587	2651.3264	0.1323	0	R.TPGGSSGGSGAAVAAGVVHVALGSDTGGSIR.I
196 – 213	1880.0327	1879.0254	1878.9952	0.0302	0	R.VGVASLSWSLDHVGPLSR.N
214 – 230	1857.8839	1856.8766	1856.8397	0.0369	0	R.NVTDAALVMQAMSGYDR.R + Oxidation (M)
214 – 230	1873.8891	1872.8818	1872.8346	0.0472	0	R.NVTDAALVMQAMSGYDR.R + 2 Oxidation (M)
214 – 231	1997.9990	1996.9917	1996.9459	0.0458	1	R.NVTDAALVMQAMSGYDRR.D
214 – 231	2014.0140	2013.0067	2012.9408	0.0659	1	R.NVTDAALVMQAMSGYDRR.D + Oxidation (M)
214 – 231	2030.0061	2028.9988	2028.9357	0.0631	1	R.NVTDAALVMQAMSGYDRR.D + 2 Oxidation (M)
231 – 254	2297.2354	2296.2281	2296.1482	0.0799	1	R.RDPGTANVAVPDMVSGIDAGVAGK.K
232 – 255	2269.2273	2268.2200	2268.1420	0.0780	1	R.DPGTANVAVPDMVSGIDAGVAGKK.I
255 – 266	1438.7508	1437.7435	1437.7616	-0.0181	1	K.KIGIPVNYYTDR.V
256 – 266	1310.6380	1309.6307	1309.6666	-0.0359	0	K.IGIPVNYYTDR.V
256 – 276	2248.2337	2247.2264	2247.1535	0.0729	1	K.IGIPVNYYTDRVAPEAAEAAK.V
267 – 276	956.3855	955.3783	955.4974	-0.1191	0	R.VAPEAAEAAK.V
277 – 284	836.2935	835.2862	835.4440	-0.1578	0	K.VAAATFEK.L
322 – 332	1321.6024	1320.5951	1320.6310	-0.0358	1	R.NSPEKFTDEVR.T
327 – 332	766.1804	765.1731	765.3657	-0.1926	0	K.FTDEVR.T
333 – 351	2019.0787	2018.0714	2018.0143	0.0572	0	R.TLLEVGAAEPAVDYVNAMR.L
333 – 351	2035.0900	2034.0827	2034.0092	0.0735	0	R.TLLEVGAAEPAVDYVNAMR.L + Oxidation (M)
383 – 403	2340.2178	2339.2105	2339.1182	0.0923	1	R.SDPFVRWEDGTVEAATAAYVR.L
389 – 403	1638.7822	1637.7749	1637.7685	0.0064	0	R.WEDGTVEAATAAYVR.L
460 – 468	925.4434	924.4361	924.5644	-0.1283	1	R.IAPVLEKVG.-

No match to: 804.1314, 826.1175, 842.0904, 1276.5887, 1335.6152, 1345.5695, 1350.6685, 1462.6929, 1652.7910, 1715.9285, 1787.9952, 1792.9497, 1903.9802, 2211.1040, 2364.1874, 2376.3228, 2419.3948, 2425.3508, 2441.3712

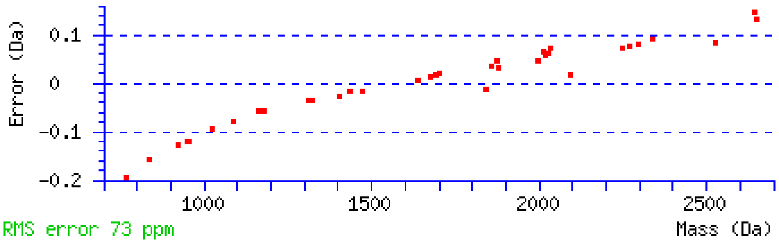

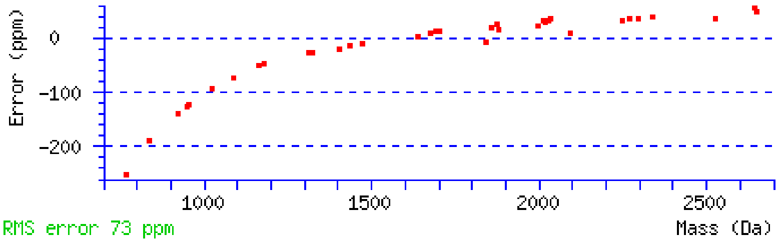

```
LOCUS       WP_011727401             468 aa            linear   BCT 18-OCT-2017
DEFINITION  Asp-tRNA(Asn)/Glu-tRNA(Gln) amidotransferase subunit GatA
            [Mycolicibacterium smegmatis].
ACCESSION   WP_011727401
VERSION     WP_011727401.1
KEYWORDS    RefSeq.
SOURCE      Mycolicibacterium smegmatis
  ORGANISM  Mycolicibacterium smegmatis
            Bacteria; Actinobacteria; Corynebacteriales; Mycobacteriaceae;
            Mycolicibacterium.
COMMENT     REFSEQ: This record represents a single, non-redundant, protein
            sequence which may be annotated on many different RefSeq genomes
            from the same, or different, species.
            COMPLETENESS: full length.
FEATURES             Location/Qualifiers
     source          1..468
                     /organism="Mycolicibacterium smegmatis"
                     /db_xref="taxon:1772"
```

```
Protein         1..468
                /product="Asp-tRNA(Asn)/Glu-tRNA(Gln) amidotransferase
                subunit GatA"
                /EC_number="3.5.1.4"
                /calculated_mol_wt=49063
Region          1..455
                /region_name="GatA"
                /note="Asp-tRNAAsn/Glu-tRNAGln amidotransferase A subunit
                or related amidase [Translation, ribosomal structure and
                biogenesis]; COG0154"
                /db_xref="CDD:223232"
```

Mascot: http://www.matrixscience.com/

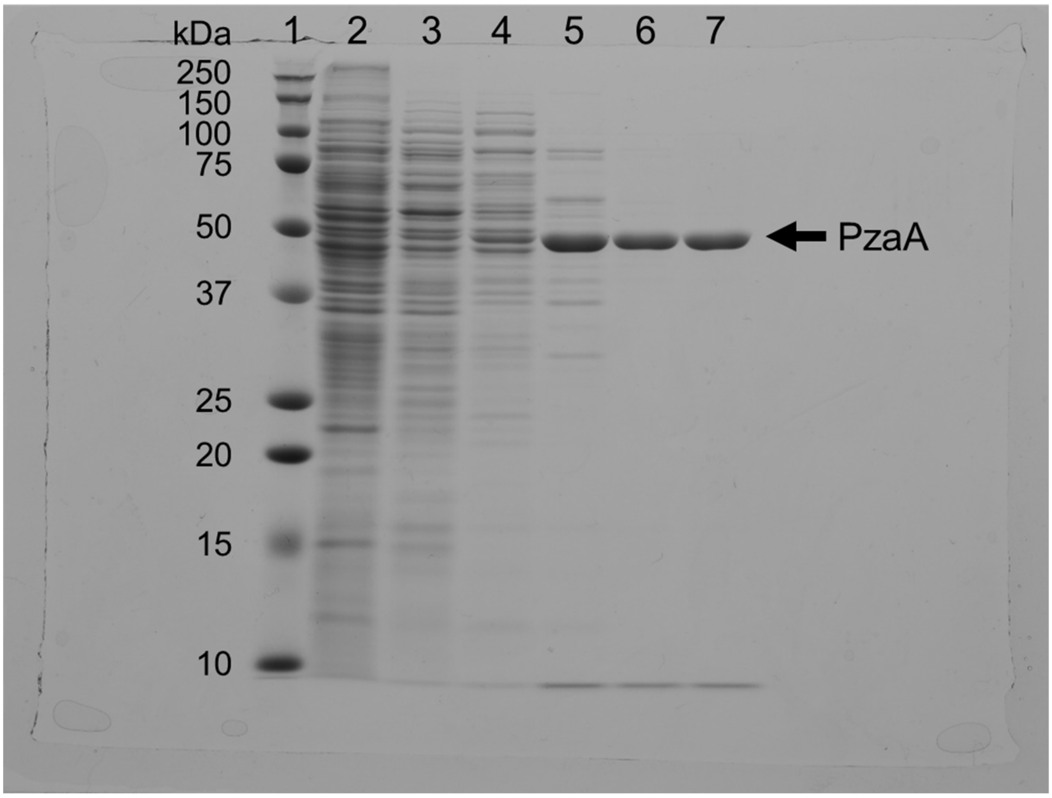

Supplementary Fig. S1. The original uncropped image of the sodium dodecyl sulfate-polyacrylamide gel electrophoresis gel shown in Figure 3. The arrow indicates the purified enzyme. Lane 1, molecular weight markers (the molecular mass of each marker in kilodaltons is indicated to the left of the gel); lane 2, cleared cell extract; lane 3, 40–60% ammonium sulfate precipitate; lane 4, pooled PzaA-containing fractions from HiTrap Q HP column chromatography; lane 5, pooled PzaA-containing fractions from HiTrap Butyl HP column chromatography; lane 6, pooled PzaA-containing fractions from Superdex 200 column chromatography; lane 7, pooled PzaA-containing fractions from Resource Q column chromatography.

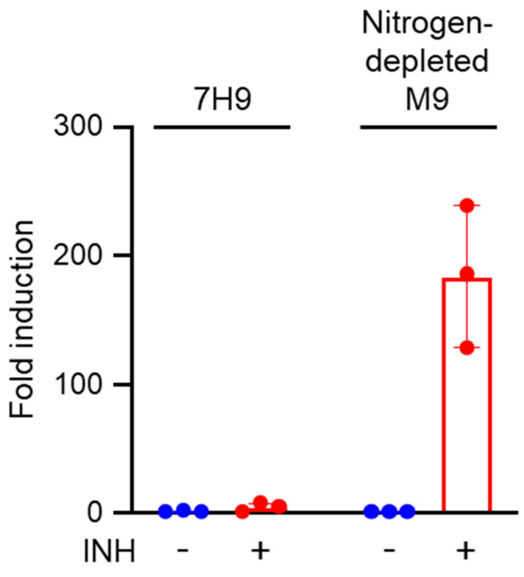

Supplementary Fig. S2. Culture medium dependence of *pzaA* transcriptional induction by isoniazid. Quantitative reverse transcription PCR was performed to measure *pzaA* mRNA levels in *Mycobacterium smegmatis* MC^{2}155. Cells were pre-cultured in the 7H9 broth for 40 h and then induced with INH (0.5 mM) in 7H9 or nitrogen-depleted M9 for 6 h. Data are expressed as mean fold-change of mRNA levels relative to the mRNA levels in cells cultivated without INH, which were set to 1.0 for each type of media. Dots and error bars indicate individual values (n = 3) and the standard deviation, respectively.

Supplementary Table S1. List of entries retrieved from BLASTP search performed with PzaA sequence as a query.

Entry no.	Description	Scientific Name	Max Score	Total Score	Query Cover	E value	Per. ident	Acc. Len	Accession
1	amidase [*Mycolicibacterium smegmatis*]	*Mycolicibacterium smegmatis*	827	827	100%	0	100	468	WP_011727401.1
2	amidase [*Mycolicibacterium smegmatis*]	*Mycolicibacterium smegmatis*	825	825	100%	0	99.79	468	WP_233044140.1
3	amidase [*Mycolicibacterium smegmatis*]	*Mycolicibacterium smegmatis*	824	824	100%	0	99.57	468	WP_003892457.1
4	amidase [*Mycolicibacterium smegmatis*]	*Mycolicibacterium smegmatis*	823	823	100%	0	99.36	468	WP_158168250.1
5	amidase [*Mycolicibacterium smegmatis*]	*Mycolicibacterium smegmatis*	822	822	100%	0	99.15	468	WP_239779559.1
6	amidase [*Mycolicibacterium smegmatis*]	*Mycolicibacterium smegmatis*	822	822	100%	0	99.36	468	WP_253803661.1
7	amidase [*Mycolicibacterium smegmatis*]	*Mycolicibacterium smegmatis*	821	821	100%	0	98.93	468	WP_058125313.1
8	Asp-tRNA(Asn)/Glu-tRNA(Gln) amidotransferase GatCAB subunit A [*Mycobacterium goodii*]	*Mycobacterium goodii*	770	770	100%	0	91.24	468	WP_073675805.1
9	amidase [*Mycobacterium goodii*]	*Mycobacterium goodii*	769	769	100%	0	91.03	468	MBU8808716.1
10	Asp-tRNA(Asn)/Glu-tRNA(Gln) amidotransferase GatCAB subunit A [*Mycobacterium goodii*]	*Mycobacterium goodii*	768	768	100%	0	91.03	468	PJK20255.1
11	amidase [*Mycobacterium goodii*]	*Mycobacterium goodii*	768	768	100%	0	91.03	468	MBU8817378.1
12	Asp-tRNA(Asn)/Glu-tRNA(Gln) amidotransferase GatCAB subunit A [*Mycolicibacterium wolinskyi*]	*Mycolicibacterium wolinskyi*	739	739	99%	0	89.08	468	WP_067859818.1
13	MULTISPECIES: Asp-tRNA(Asn)/Glu-tRNA(Gln) amidotransferase GatCAB subunit A [*Mycolicibacterium*]	*Mycolicibacterium wolinskyi* *Mycolicibacterium goodii*	736	736	99%	0	88.87	468	WP_085147449.1
14	amidase [*Mycobacterium* sp. AZCC_0083]	*Mycobacterium* sp. AZCC_0083	715	715	99%	0	86.7	469	WP_184445528.1
15	amidase [*Mycolicibacterium mengxianglii*]	*Mycolicibacterium mengxianglii*	700	700	99%	0	84.27	469	WP_199254090.1
16	amidase [*Mycolicibacterium vaccae*]	*Mycolicibacterium vaccae*	699	699	99%	0	83.91	468	WP_003932556.1
17	amidase [*Mycolicibacterium mengxianglii*]	*Mycolicibacterium mengxianglii*	699	699	99%	0	84.27	469	WP_197378416.1
18	amidase [*Mycolicibacterium litorale*]	*Mycolicibacterium litorale*	697	697	99%	0	83.62	468	WP_185293653.1
19	amidase [*Mycolicibacterium baixiangningiae*]	*Mycolicibacterium baixiangningiae*	697	697	99%	0	83.19	468	WP_193045577.1
20	amidase [*Mycobacterium doricum*]	*Mycobacterium doricum*	688	688	99%	0	81.9	468	WP_085188850.1

Entry no.	Description	Scientific Name	Max Score	Total Score	Query Cover	E value	Per. ident	Acc. Len	Accession
21	Asp-tRNA(Asn)/Glu-tRNA(Gln) amidotransferase GatCAB subunit A [*Mycobacterium* sp. AT1]	*Mycobacterium* sp. AT1	686	686	99%	0	82.4	468	WP_079924399.1
22	amidase [*Mycobacterium hodleri*]	*Mycobacterium hodleri*	682	682	99%	0	82.19	468	WP_142551520.1
23	amidase [*Mycolicibacterium baixiangningiae*]	*Mycolicibacterium baixiangningiae*	682	682	97%	0	82.93	461	WP_226863433.1
24	amidase [*Mycolicibacterium baixiangningiae*]	*Mycolicibacterium baixiangningiae*	682	682	97%	0	82.71	461	WP_231644238.1
25	amidase [*Mycolicibacterium* sp. P1-18]	*Mycolicibacterium* sp. P1-18	677	677	99%	0	81.55	468	WP_149484003.1
26	amidase [*Mycolicibacterium murale*]	*Mycolicibacterium murale*	672	672	98%	0	81.21	465	WP_193491350.1
27	amidase [*Mycobacterium* sp. djl-10]	*Mycobacterium* sp. djl-10	672	672	98%	0	81.21	465	ANW63176.1
28	amidase [*Mycobacterium* sp. MS1601]	*Mycobacterium* sp. MS1601	672	672	98%	0	82.07	465	WP_083736400.1
29	Asp-tRNA(Asn)/Glu-tRNA(Gln) amidotransferase GatCAB subunit A [*Mycolicibacterium canariasense*]	*Mycolicibacterium canariasense*	671	671	99%	0	80.51	468	WP_062655887.1
30	amidase [*Mycobacterium scrofulaceum*]	*Mycobacterium scrofulaceum*	448	448	97%	1.00E-152	55.82	482	WP_067282891.1
31	amidase [*Mycobacterium scrofulaceum*]	*Mycobacterium scrofulaceum*	447	447	97%	2.00E-152	55.82	482	WP_067309787.1
32	Asp-tRNA(Asn)/Glu-tRNA(Gln) amidotransferase GatCAB subunit A [*Mycobacterium asiaticum*]	*Mycobacterium asiaticum*	425	425	96%	4.00E-144	53.44	463	WP_036355272.1
33	Asp-tRNA(Asn)/Glu-tRNA(Gln) amidotransferase GatCAB subunit A [*Mycobacterium asiaticum*]	*Mycobacterium asiaticum*	423	423	96%	5.00E-143	52.99	463	WP_065138022.1
34	amidase [*Mycobacterium simiae*]	*Mycobacterium simiae*	422	422	96%	1.00E-142	54.55	463	WP_061559226.1
35	Asp-tRNA(Asn)/Glu-tRNA(Gln) amidotransferase GatCAB subunit A [*Mycobacterium paraffinicum*]	*Mycobacterium paraffinicum*	422	422	97%	2.00E-142	54.07	481	WP_073876963.1
36	amidase [*Mycobacterium tuberculosis* variant microti OV254]	*Mycobacterium tuberculosis* variant microti OV254	418	418	96%	3.00E-141	54.1	463	PLV44878.1
37	amidase [*Mycobacterium sherrisii*]	*Mycobacterium sherrisii*	417	417	97%	9.00E-141	52.75	471	WP_069402088.1
38	amidase [*Mycobacterium simiae*]	*Mycobacterium simiae*	417	417	95%	1.00E-140	54.24	468	WP_232069455.1
39	amidase [*Mycobacterium simiae*]	*Mycobacterium simiae*	415	415	95%	7.00E-140	54.02	468	WP_231382624.1
40	amidase [*Mycobacterium saskatchewanense*]	*Mycobacterium saskatchewanense*	404	404	96%	2.00E-135	53.88	476	WP_085256999.1
41	amidase [*Mycobacterium sherrisii*]	*Mycobacterium sherrisii*	379	379	84%	9.00E-127	53.05	410	WP_244899560.1

Supplementary Table S2. List of primers used in this study.

Primers	Sequence
For constructing the pzaA *deletion mutant*	
Dis_pzaA_1	5′ CGGGCCGAATTCGACCGGGGTTTG 3′
Dis_pzaA_2	5′ CTCGACCGGTGAGACCTCCTTG 3′
Dis_pzaA_3	5′ GTCTCACCGGTCGAGTTCGGATATGCCTTG 3′
Dis_pzaA_4	5′ GCGCGGAAGCTTAGCGCCTGC 3′
For constructing the pMV261-pzaA *plasmid*	
Exp_PzaA_F	5′ GAATCTGCAGAGTGAAAAGGAAC 3′
Exp_PzaA_R	5′ GTGCCAAGCTTCATGTCAGCCCAC 3′
For constructing the pMyC-pncA *plasmid*	
Exp_PncA_F	5′ AGGGAGTCCACCATGCGTGCACTGATTGTCGTC 3′
Exp_PncA_R	5′ TGGTGGTGCGAAGCTTCAGCTGATGTCACTCCC 3′
For quantitative reverse transcription PCR	
qRT_sigA_F	5′ AAAAACCATCTGCTGGAGGC 3′
qRT_sigA_R	5′ AGAACTTGTAGCCCTTGGT 3′
qRT_pzaA_F	5′ GGTCAACTACTACACCGACC 3′
qRT_pzaA_R	5′ GATCTCGACCTCGACAAGTT 3′

Recognition sites for restriction enzymes are underlined.
